# Successful treatment of enterocutaneous fistula after esophagectomy with scopolamine ointment and negative pressure wound therapy: a case report

**DOI:** 10.1186/s40792-020-00938-2

**Published:** 2020-07-22

**Authors:** Shigemasa Suzuki, Ryusuke Aihara, Takashi Ooki, Naoki Matsumura, Wataru Wada, Akira Mogi, Yasuo Hosouchi, Yasuji Nishida, Makoto Sakai, Makoto Sohda, Hiroshi Saeki, Ken Shirabe

**Affiliations:** 1 Department of Surgery, Gunma Prefecture Saiseikai Maebashi Hospital, 564-1, Kamishinden-machi, Maebashi, Gunma 371-0821 Japan; 2 Department of General Surgical Science, Graduate School of Medicine, Gunma University, Maebashi, Japan

**Keywords:** Intractable enterocutaneous fistula, Esophagectomy, Scopolamine ointment, Negative pressure wound therapy

## Abstract

**Background:**

Despite improved surgical techniques and perioperative management, anastomotic leakage (AL) after esophageal cancer surgery remains a potential complication. In most cases, spontaneous healing upon proper drainage is observed, but sometimes, AL results in intractable enterocutaneous fistulas. We here report a case of intractable enterocutaneous fistula caused by post-esophagectomy AL and successfully treated by scopolamine ointment and negative pressure wound therapy (NPWT).

**Case presentation:**

A 77-year-old man underwent thoracoscopic subtotal esophagectomy with 3-field lymph node dissection, followed by gastric tube reconstruction through the posterior mediastinal route. On the 6th postoperative day, AL was identified, forming an enterocutaneous fistula. Initially, conservative treatment was performed, but the fistula failed to close. We hypothesized that the substantial amount of exudate might be hampering fistula closure. Scopolamine ointment was used to reduce the amount of fluid. NPWT was also initiated to promote wound healing. Approximately 3 weeks after the beginning of the treatment, the fistula closed; oral intake became possible, and the patient was discharged from the hospital without any symptoms.

**Conclusions:**

The combination of scopolamine ointment and NPWT may be regarded as one effective treatment option for intractable enterocutaneous fistula due to AL after esophagectomy.

## Background

Anastomotic leakage (AL) is one of the major complications of esophageal cancer surgery, with a frequency of 13.3% according to the Japanese National Clinical Database [1]. In most cases, spontaneous healing is observed after conservative treatment, which may include fasting and proper drainage; however, intractable enterocutaneous fistulas sometimes develop. Large amounts of fistula discharge significantly reduce the patient’s quality of life and prolong the fasting period and hospitalization.

Negative pressure wound therapy (NPWT) is a relatively new treatment, which promotes healing by sealing the wound surface [2]. The use of NPWT for enterocutaneous fistula due to post-esophagectomy AL has rarely been reported. Here, we report a case of postoperative fistula successfully managed by a combination of scopolamine ointment and NPWT.

## Case presentation

**Fig. 1:**
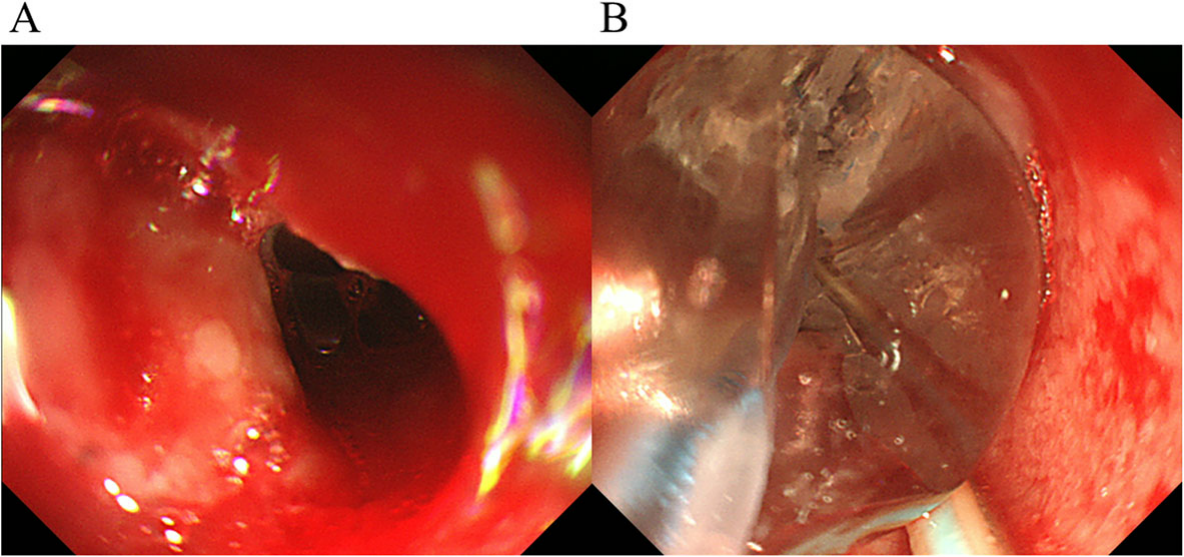
**a** Endoscopic examination confirmed the presence of anastomotic stenosis. **b** Endoscopic balloon bougie was performed on the 58th POD

**Fig. 2:**
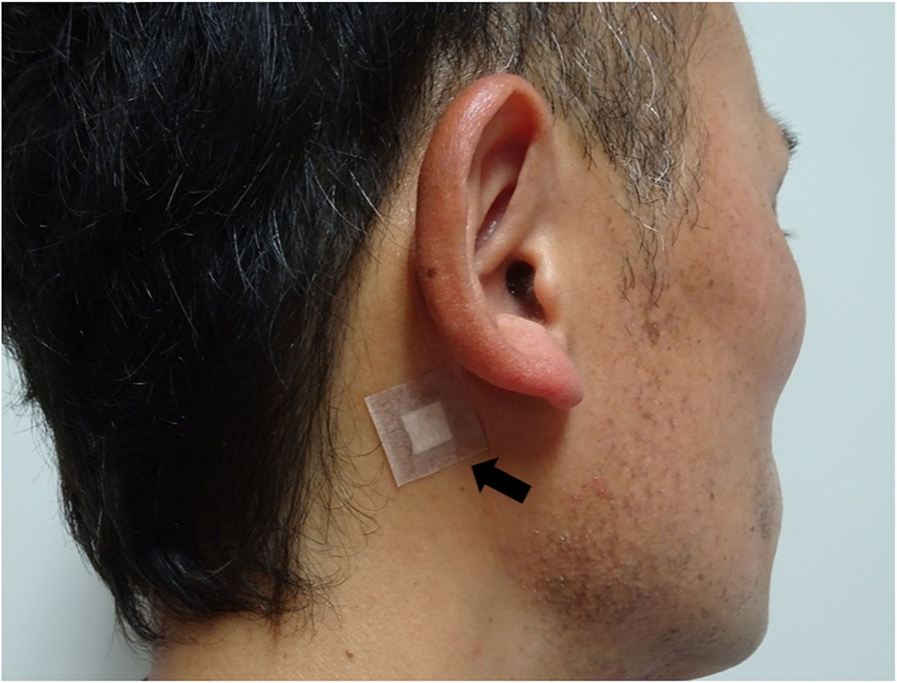
Position in which the scopolamine patch was attached. A small amount of scopolamine ointment was applied near the papillary process in the posterior auricle

**Fig. 3:**
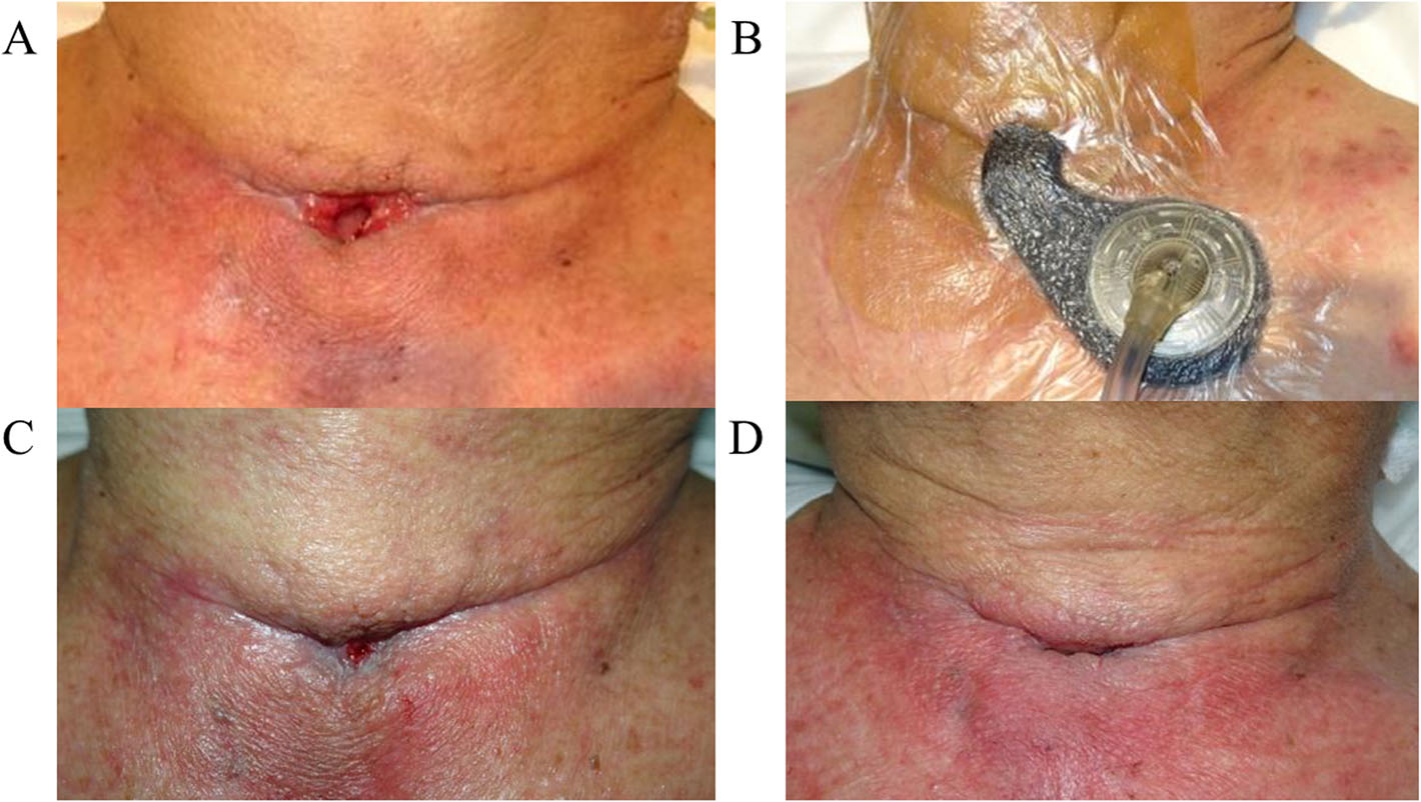
**a** Appearance of the cervical wound on the 61st POD. Exudate constantly emerging from the fistula caused peripheral dermatitis. **b** NPWT was initiated for continuous aspiration of exudate and skin protection. **c** Appearance of the cervical wound on the 73rd POD (13th day after NPWT initiation). Granulation is in development, the skin closes in, and the fistula is shrinking. **d** Appearance of the cervical wound on the 81st POD (21st day after NPWT initiation). The fistula had closed, and no exudate was observed

A 77-year-old man underwent esophagectomy for thoracic esophageal cancer with 3-field lymph node dissection, followed by gastric tube reconstruction through the posterior mediastinal route. The reconstruction was performed in the cervical region with end-to-side anastomosis using an automatic anastomotic device. Eventually, the length of the residual esophagus was about 3 cm. On the 6th postoperative day (POD), redness was observed around the cervical wound. The wound was partially opened, and AL discharge was observed. Conservative treatment, including fasting, drainage of defective granulation, and tube feeding, was initiated; however, a large amount of exudate continued to come out, producing an enterocutaneous fistula. On the 29th and 43rd POD, we attempted to close the fistula by suturing, but it soon broke apart. On the 57th POD, we injected Dermabond® into the fistula, but to no avail. On the 58th POD, because it is considered that the stenosis of the anastomosis is an obstacle to the healing of the fistula, endoscopic balloon bougie to enlarge the size of anastomosis was performed (Fig. 1a, b). However, the exudate was never reduced. Therefore, we speculated that the large amount of exudate incessantly flowing out might be obstructing fistula closure. First, we aimed to reduce the flow through the fistula using scopolamine ointment. On the 61st POD, a small amount of scopolamine ointment was applied near the papillary process in the posterior auricle twice a day (Fig. 2). In Japan, transdermal scopolamine is not commercially available, and therefore, it was prepared in our hospital; its use was approved by the Ethics Review Committee in our hospital for off-label use. Although there was concern about anticholinergic side effects, only mild thirst was recognized. Simultaneously, NPWT was initiated to promote wound healing (Fig. 3a, b). A vacuum-assisted closure treatment system (KCI International, San Antonio, TX, USA) was used as a negative pressure maintenance device, and the polyurethane foam was replaced every 72 h. The negative pressure was appropriately adjusted to 100 to 150 mmHg. Sometimes, the negative pressure stopped. This issue was addressed by adjusting the suction pressure and appropriately improving the shape of the polyurethane foam. Gradually, good granulation appeared, and the fistula shrank (Fig. 3c). Approximately 3 weeks after the beginning of the treatment, the fistula had closed, and oral intake was possible (Fig. 3d). The patient was discharged from the hospital on the 97th POD with no symptoms.

## Discussion

Enterocutaneous fistulas complicating esophageal cancer surgery can become intractable due to substantial exudate, which constantly exposes the surrounding skin to strong irritation and impairs granulogenesis. Also, a large amount of fistula exudate causes considerable pain [3, 4]. For intractable enterocutaneous fistulas arising due to AL, a minimally invasive approach should be considered first. Therefore, surgical treatments such as pedicled myocutaneous flap filling [5] are not among the first-choice treatments. The usefulness of covered stents for fistula closure after esophagectomy has been reported [6, 7]. However, in this case, since the anastomosis was located in the neck, endoscopic placement of the stent was not suitable. Although over-the-scope clip system was reported as one of the treatment options for anastomotic fistula [8], it was technically difficult to grasp all layers of the digestive tract and was abandoned. Intrathecal injection of fibrin glue or cyanoacrylate has been reported as useful treatment for intractable fistulas [9, 10]. The mechanism of action of fibrin glue involves fibroblasts invading and proliferating in the fibrin matrix adhering to the tissue, resulting in tissue repair with angiogenesis. However, it takes several minutes for fibrin to form a stable cross-link and adhere to the tissue, and the adhesion and fixation are weak. Therefore, this method appeared unsuitable for this case, where exudate was constantly emerging, and the pressure was high. Cyanoacrylate preparations have the advantages of quick and high-strength adhesion [11], and so we attempted to close the fistula using Dermabond®, which, however, proved ineffective.

As the excessive exudate was suspected to be one of the causes of delayed wound healing, we first aimed to reduce the amount of exudate. Although drug therapy options include the use of anticholinergic drugs, both oral and intravenous administration may lead to adverse events such as blurred vision, miosis, palpitation, hot flushes, dizziness, and constipation. Meanwhile, scopolamine is known to be transdermally absorbed [12]. Regarding skin permeability, the posterior part of the ear shows high skin permeability. In the USA, a scopolamine patch is an anticholinergic preparation approved for prevention of motion sickness and is commercially available [13]. Scopolamine patches have also been reported to suppress saliva production in cancer patients [14]. In Japan, such patches are not commercially available, and so one was prepared in our hospital in addition to a hydrophilic ointment so that the concentration of scopolamine hydrobromide hydrate was 5%. In our case, from the day of application, the amount of saliva decreased to about half, and only a slight degree of mouth dryness was observed.

NPWT is a physical therapy in which a negative pressure is continuously or intermittently applied to a wound in a closed environment to promote the formation of granulation tissue, adjust the wound bed, and hence facilitate wound healing. It is widely used for acute wounds, such as those that cannot be closed temporarily; open wounds on amputated limbs; and chronic wounds such as pressure ulcers and diabetic foot ulcers [15–17]. Endoscopic vacuum-assisted closure treatment is increasingly used for intrathoracic leakage after esophagectomy [18]. On the other hand, there have been few reports of percutaneous NPWT for management of enterocutaneous fistula due to AL after esophagectomy [19]. We speculated that NPWT would be fully applicable to postoperative enterocutaneous fistulas, such as the one in the present case, and would fit the criterion of a minimally invasive approach. It is also thought that a synergistic effect was obtained in the process of fistula closure by using scopolamine ointment in combination. The reduction in the exudate enabled protection of the wound, and granulation was promoted by the continuous negative pressure. A problem with the use of NPWT in intestinal tract communication is the risk of intestinal necrosis associated with continuous negative pressure [20], possibly due to the negative pressure impairing the microcirculation [21]. Therefore, the negative pressure was carefully and gradually initiated in the present case. Meanwhile, this treatment did not require frequent gauze replacement due to continuous drainage and improved the condition of the surrounding skin, not only promoting wound healing but also significantly reducing patient discomfort.

To our knowledge, there is no general definition of the duration of an intractable fistula. In our case, although post-esophagectomy AL was observed, the drainage and the general condition were reasonably good with no serious complications. Therefore, the next treatment was delayed and the hospitalization period became quite long. We suggest that AL that does not improve within 1 month at the latest should be considered as intractable fistula, and in such cases, a combination treatment with scopolamine ointment and NPWT may prove a successful treatment strategy.

## Conclusion

The combination of scopolamine ointment and NPWT may be regarded as one effective treatment option for intractable enterocutaneous fistula due to AL after esophagectomy.

## Data Availability

The data supporting the conclusions of this article are included within the article.

## Abbreviations

AL: Anastomotic leakage
NPWT: Negative pressure wound therapy
POD: Postoperative day

## References

[1] Takeuchi H, Miyata H, Gotoh M (2014). A risk model for esophagectomy using data of 5354 patients included in a Japanese nationwide web-based database. Ann Surg.

[2] Argenta LC, Morykwas MJ (1997). Vacuum-assisted closure: a new method for wound control and treatment: clinical experience. Ann Plast Surg.

[3] Martinez JL, Luque-de-Leon E, Mier J (2008). Systematic management of postoperative enterocutaneous fistulas: factors related to outcomes. World J Surg.

[4] Evenson AR, Fischer JE (2006). Current management of enterocutaneous fistula. J Gastrointest Surg.

[5] Hayashi K, Ando N, Ozawa S (1999). Gastric tube-to-tracheal fistula closed with a latissimus dorsi myocutaneous flap. Ann Thorac Surg.

[6] Gelbmann CM, Ratiu NL, Rath HC (2004). Use of self-expandable plastic stents for the treatment of esophageal perforations and symptomatic anastomotic leaks. Endoscopy.

[7] Hunerbein M, Stroszczynski C, Moesta KT, Schlag PM (2004). Treatment of thoracic anastomotic leaks after esophagectomy with self-expanding plastic stents. Ann Surg.

[8] Kobara H, Mori H, Fujihara S (2017). Outcomes of gastrointestinal defect closure with an over-the-scope clip system in a multicenter experience: an analysis of a successful suction method. World J Gastroenterol.

[9] Nakano Y, Takao T, Morita Y (2019). Endoscopic plombage with polyglycolic acid sheets and fibrin glue for gastrointestinal fistulas. Surg Endosc.

[10] Ojima T, Nakamura M, Nakamori M (2018). Endoscopic treatment of esophageal fistulas after esophagectomy with injection of an alpha-cyanoacrylate monomer: a phase II study. Endosc Int Open.

[11] Nasralla M, Fahad AA (2019). Successful radiological embolization of a low output jejunal enterocutaneous fistula using a cyanoacrylate and lipiodol mixture. Radiol Case Rep.

[12] Clissold SP, Heel RC (1985). Transdermal hyoscine (Scopolamine). A preliminary review of its pharmacodynamic properties and therapeutic efficacy. Drugs.

[13] Richardson CT, Feldman M (1986). Effects of transdermal scopolamine, alone or in combination with cimetidine, on total 24 hour gastric acid secretion in patients with duodenal ulcer. Gut.

[14] Tassinari D, Poggi B, Fantini M (2005). Treating sialorrhea with transdermal scopolamine. Exploiting a side effect to treat an uncommon symptom in cancer patients. Support Care Cancer.

[15] Banwell PE, Musgrave M (2004). Topical negative pressure therapy: mechanisms and indications. Int Wound J.

[16] Wackenfors A, Sjogren J, Gustafsson R (2004). Effects of vacuum-assisted closure therapy on inguinal wound edge microvascular blood flow. Wound Repair Regen.

[17] Kaushik D, Joshi N, Kumar R (2017). Negative pressure wound therapy versus gauze dressings for the treatment of contaminated traumatic wounds. J Wound Care.

[18] Min YW, Kim T, Lee H (2019). Endoscopic vacuum therapy for postoperative esophageal leak. BMC Surg.

[19] Endara SA, Teran FJ, Serrano AJ (2018). Esophagocoloplasty fistula successfully treated with vacuum-assisted closure. J Surg Case Rep.

[20] Rao M, Burke D, Finan PJ, Sagar PM (2007). The use of vacuum-assisted closure of abdominal wounds: a word of caution. Colorectal Dis.

[21] Lindstedt S, Malmsjo M, Hansson J (2012). Microvascular blood flow changes in the small intestinal wall during conventional negative pressure wound therapy and negative pressure wound therapy using a protective disc over the intestines in laparostomy. Ann Surg.

